# Neurologic long term outcome after drowning in children

**DOI:** 10.1186/1757-7241-20-55

**Published:** 2012-08-15

**Authors:** Pertti K Suominen, Raisa Vähätalo

**Affiliations:** 1 Department of Anaesthesia and Intensive Care, Children’s Hospital, Helsinki University Central Hospital, Stenbäckinkatu 9, FIN-00029 HUS, Helsinki, Finland

**Keywords:** Drowning, Cardiopulmonary resuscitation, Children, Outcomes, Health related quality of life

## Abstract

Drowning is a major source of mortality and morbidity in children worldwide. Neurocognitive outcome of children after drowning incidents cannot be accurately predicted in the early course of treatment. Therefore, aggressive out-of-hospital and in-hospital treatment is emphasized. There are "miracle" cases after long submersion times that have been reported in the medical literature, which mostly concern small children. However, many of the survivors will remain severely neurologically compromised after remarkably shorter submersion times and will consequently be a great burden to their family and society for the rest of their lives. The duration of submersion, the need of advanced life support at the site of the accident, the duration of cardiopulmonary resuscitation, whether spontaneous breathing and circulation are present on arrival at the emergency room are important factors related to survival with mild neurological deficits or intact function in drowned children. Data on long-term outcome are scarce. The used outcome measurement methods and the duration of follow-up have not been optimal in most of the existing studies. Proper neurological and neurophysiological examinations for drowned children are superior to outcome scales based chart reviews. There is evidence that gross neurological examination at the time of discharge from the hospital in young children does not reveal all the possible sequelae related to hypoxic brain injury and thus long-term follow-up of drowned resuscitated children is strongly recommended.

## Introduction

It is increasingly recognized that the assessment of health related quality of life (HRQoL) should become a standard of care in children after trauma and cardiac arrest f [1,2]. In contrast, there have been numerous attempts to determine different predictors of neurologic outcome at hospital discharge after drowning accidents [3-12]. However, little is known about long-term neurocognitive outcome after a drowning incident. The neurological status of survivors is often retrospectively evaluated using hospital records and also the Pediatric Overall Performance Category Scale (POPC) at discharge and at 1-year after the incident. These evaluations are currently recommended by the Utstein guidelines for research on drowning [13-16]. Pediatric drowning victims may have grossly intact neurological examinations at discharge from the hospital, but the long-term cognitive sequelae may not manifest until the child enters school [17,18].

The aims of this review are a) to report the main factors related to the outcome of drowned children and b) to present existing evidence of long-term neurologic outcome. The latter findings were obtained from reviewed studies that report the outcome of children after extended follow-up periods.

## Materials and methods

Bibliographic MEDLINE and PUBMED databases were searched for English language literature using medical subject headings: 1) drowning or near drowning; 2) submersion and immersion; 3) children; 4) outcome and 5) quality of life. The search was done from inception until March 2012. We also searched previously published literature for additional references. We included all the studies that report the outcome of drowned children after extended follow-up periods. We excluded studies without any follow-up of drowned children after the hospital discharge. One case report was included, because it was the only study published with an adequate follow-up time [18].

### Definition of drowning

A review by Papa et al. found a total of 35 different definitions to describe drowning incident and 20 different outcome measures [19]. The variability of these definitions and outcomes makes it very difficult to assess and draw conclusions from the existing literature. Therefore, in 2002 consensus experts agreed upon a definition that includes both fatal and non-fatal drowning cases. The following definition was adopted: “Drowning is the process of expiring respiratory impairment from submersion/immersion in liquid” [20]. Implicit in this definition is that liquid/air interference occurs at the entrance of the airways of the victim, which prevents the victim from breathing air [19,20]. A victim can be rescued at any time during the drowning process and may not require any intervention. On the other hand, victims may receive appropriate resuscitative measures, in which case the drowning process is interrupted [13]. Drowning outcomes should be classified according to the following categories: death, morbidity or no morbidity [13,20].

### Epidemiology

Drowning is the fifth leading cause of accidental deaths in the United States. It is also the second leading cause of accidental deaths among children aged 1–14 years in the US [21]. Although the incidence of drowning in children younger than 15 years of age is 1.1/100 000, the incidence/ of drowning is highest among 0–4 year-old children [21-23]. Drowning studies are based on national or state statistics, national drowning rescue organizations databases and ICD codes at hospital discharge [21,24,25]. Most of these databases are not designed for research purposes and so they may not include all the drowning cases that actually occurred. Furthermore, the outcomes of the victims in particular are not very reliably reported nor are they very robust as cases are categorized as either survive or die [24].

Some children with a witnessed short submersion time will begin breathing and regain conscious after they have been removed from the water and they may also have received some rescue mouth-to-mouth breaths. Some of these minor cases may not have received hospital treatment and thus may not be recorded in the national statistics. At the other end of the spectrum, are children brought to the hospital who have had long submersion and rescue times, who consequently suffer from severe anoxic brain injury and who are subsequently discharged to another institution. When some of these patients decease months or years after the accident due to pneumonia or some other causes, they are not necessary included in the drowning statistics *per se*: instead they are often reported in studies as survivors of drowning.

There are also differences in the quality of out-of-hospital care and the decision making at the site of the accident. In many European countries, physician staffed emergency units are able to pronounce a drowned patient dead with or without cardiopulmonary resuscitation (CPR). Such a procedure is in contrast to that used by emergency medical services (EMS) units that are staffed by emergency technicians or paramedics who usually transport all the victims with ongoing CPR to the emergency room (ER). Therefore, the outcome of drowned patients treated in different hospitals and in different countries may not be comparable.

The ratio between fatal and non-fatal cannot be reliably determined because of the above-mentioned reasons in reporting the incidences of drowning and because of the inconsistencies in the terms used for drowning. Nevertheless, it has been estimated that the numbers of non-fatal drownings are two to four times higher than the numbers of fatal drownings [8,25-27].

Most of the drowning accidents in children occur in natural bodies of water [8,20,27,28]. However, drownings often occur in bathtubs for infants and in private pools for toddlers during brief lapses in adult supervision [26,27,29]. Proper safety barrier/childproof fencing of private pools and garden ponds, and water safety training to children at a young age are instrumental in reducing the risk for drowning [26,28,29]. Continuous adult supervision in or near water can prevent many of these deaths, especially drownings in bathtubs and pools [26,27,29].

### Hypoxic ischemic brain injury

Organs such as the brain, lungs and kidneys are mainly affected by drowning accidents. Treatment of pulmonary complications depend on the lung injury that was incurred during aspiration and also the bacteria that was aspirated [25]. Some patients may develop adult respiratory disease syndrome (ARDS) and may even need ECMO to survive. However, the long-term outcome of survived drowning victims depend mainly on the severity of the initial ischemic brain insult, the effectiveness of immediate resuscitation with subsequent transfer to the ER, and also on the post-resuscitation management in the intensive care unit [30,31]. The most susceptibility areas to ischemic injuryare vascular end zones, hippocampus, insular cortex, and basal ganglia. With greater severity of hypoxic-ischemia, more extensive and global neocortical injury will occur [23].

Important predictors for survival itself either with mild or severe neurological deficits include: the duration of submersion, the need of advanced life support at the site of the accident, the duration of CPR, and the establishment of spontaneous breathing and circulation on arrival to the ER [3,5,8,10,16,27,32]. Submersion time mainly determines the level of hypoxic-ischemic injury but it is at best an estimate given in an extremely stressful situation. It has been shown that a prolongation of submersion over 5 –10 min worsens the prognosis considerably [5,8]. Many other predictors of survival that have been reported in the literature are mainly consequences of the duration of the primary insult of CPR and the quality of the treatment the patient has received before or after the arrival to the ER. Laboratory values such as severe acidotic pH-values, high blood sugar and lactate are usually signs of a long submersion and resuscitation time and therefore they are signs of poor outcome except in hypothermic children drowned in icy water [7,10,27,32-36]. There are insufficient data on biochemical markers such as neuron-specific enolase (NSE) or serum astroglial protein (S-100B) in children after cardiac arrest to help outcome prediction [31].

There is no entirely reliable algorithm of clinical signs or investigations that allow a definitive prognosis but the combination of careful repeated observations and examinations will give accurate information to advise on management [30,31]. Clinical assessment in the PICU is also often compromised by factors that include: sedation, neuromuscular blockade, ventilation, hypothermia and inotropic management [30,31]. The presence of any motor activity and pupillary reactivity noted on arrival to the ER could significantly discriminate between survivors and fatalities, but could not discriminate between intact and vegetative survivors [6]. Successful control of intracranial pressure (ICP) and cerebral perfusion pressure (CPP) did not ensure intact survival and a sustained late intracranial hypertension was more likely to be a sign of irreversible brain damage [6,35,37]. The duration of consciousness after drowning when good recovery is still considered possible seem to vary in the literature. In a study by Bratton et al. all satisfactory survivors were sufficiently awake and had spontaneous, purposeful movements and normal brain-stem function as early as 24 hours after the drowning event [11]. In comparison, the findings of Bell et al. show that all children who made good recoveries regained consciousness within a two-week period [6].

Repeated or continuous electroencephalogram (EEG) may provide useful information to assist the differentiation between patients with good and poor neurological outcome. Reactivity to auditory and painful stimulations is a more important sign of good prognosis than the dominant EEG frequency alone [30,31,38]. A bad outcome can be associated with burst-suppression, generalized suppression, status epilepticus, and nonreactivity [31,38]. Somatosensory evoked potentials are valuable in assessing prognosis and they are also less susceptible than EEG tosedation and metabolic factors. However, the accuracy in predicting neurological outcome is still not very good [30,31].

The increased use of neuroimaging techniques can add valuable information, in particular brain magnetic resonance imaging (MRI) shows characteristic patterns depending on the severity of the injury and also the timing of imaging [30,31]. The degree of edema and brain swelling is better seen by MRI than by computer tomography (CT) scans, therefore CT scans are not widely used for early outcome prediction [31]. In the European resuscitation council guidelines for resuscitation, it is recommended to consider induced hypothermia for 12 – 24 hours for children who remain comatose following resuscitation, although there is no strong scientific evidence in the literature to support this treatment of children [39].

### Studies on long-term outcome

A good functional outcome after a drowning accident is vital because severe neurological injury will incur a great burden to the victim’s family and to society as a whole. Data on long-term outcome are scarce. We were able to find only six articles that included neurologic follow-up data on solely drowned pediatric patients from the existing literature. In addition, one case report and one case series were included in the review (Table 1) [6,8,10,11,18,33,40,41]. 

**Table 1:** Studies on long term survival in children after drowning accident

**Study**	**Design Patient population**	**Patients (N)**	**Age**	**Patient selection**	**Follow up period**	**Assessment tool**	**Conclusion**
**Pearn 1977**[40]	Prospective 1971-75	54	Not reported	Freshwater immersion accidents in which consciousness was lost	Median 23 months (3–60 months)	Neurological and neuropsychological testing	95% of children survived neurologically normal
**Kruus et al. 1979**[10]	Prospective 1971-76	30	Median 4 years	Drowned children of whom 28/30 had been resuscitated	Median 22 months (6–58 months)	Neurological and neuropsychological testing of 17 survivors	13 children with slight neurological deficit and 4 with severe deficit
**Bell et al. 1984**[6]	Prospective 1979-83	49	8-154 months	Drowned children who had been resuscitated by EMS personnel	8-40 months	Neurological and neuropsychological testing of 7 apparently intact survivors	Long-term survivors had nearly normal levels of cognitive function
**Bratton et al. 1994**[11]	Retrospective 1986-91	44	Median 28 months (8 mo-14 yrs)	Children admitted to PICU after warm drowning, 43/44 received CPR at the scene	Minimum of 6 months	A discussion with child’s physician or chart review	17/44 (39%) had normal functioning or mild neurological deficit
**Suominen et al 1997**[8]	Retrospective 1985-94	48	Median 3.7 years (0.8-15.0 years)	Drowned children who had received ALS at the scene	1 year	Chart review with POPC	29/48 (60%) had normal functioning or mild neurological deficit
**Hughes et al. 2002**[18]	Case report 1986	1	2.5 years	Neuropsychological recovery after 66 min submersion, CPR and CPB	12 years	Neuropsychological testing, neuroimaging	Cognitive difficulties and global memory impairment in follow-up
**Suominen et al. 2010**[33]	Retrospective 1994-2008	9	Median 3.7 years (0.8-15.0 years)	Hypothermic drowning victims treated with CPB	3 years	Neurological and neuropsychological testing	One survived with mild to moderate neurological deficit
**Suominen et al.2011**[41]	Questionnaire	29	Median 3.0 (range 1.2-15.7) years	Drowned children who had been resuscitated either bystanders and/or EMS personnel	Median 10.3 years (1.8-21.8 years)	Mailed HRQoL questionnare	Good HRQoL in most of the long term survivors

Abbreviations: *CPR*, Cardiopulmonary resuscitation; *ALS*, advanced life support; *POPC*, Paediatric Overall Performance Category Scale; *HRQoL*, Health-related quality of life.

Pern studied 56 children with freshwater immersion accidents in the Brisbane area of Australia, in which consciousness was lost in the water and which also necessitated a subsequent admission to hospital [40]. The mean estimated submersion time was 3.7 min (range 0.5 – 10 min). Fifty-four children were re-examined medically and psychometrically. Of these 54 children, 52 were completely normal. The other two patients had severe neurologic sequelae. The median IQ of the survivors was 110 (range 90–137), which is higher than that of the general population. There is a suggestion that visualmotor (performance) skills are particularly vulnerable to freshwater immersion hypoxia. In 20 percent of survivors subscale disparities between verbal and performance skills exceeded 15 IQ-points. No long-term emotional or personality disorders were encountered [40].

In an early study Kruus et al. reported on 30 children patients who were treated in the Children's Hospital in Helsinki, Finland, after a drowning accident. The submersion time was not known 11 patients, but in other patients it was estimated to be between less than 5 to 20 minutes. All except two out of 30 patients needed CPR after the accident. Thirteen children (43%) died in the hospital. The surviving 17 children underwent neurological, neurophysiological and psychological examination 6 – 58 months after the accident. Four of the 17 surviving children were tetraplegic, unable to speak and had convulsions. Thirteen children (43%) had slight neurological or psychological signs. Their median general IQ was 96 (range 88–115), except for two of these 13 children whose respective IQs were 48 and 76 [10].

In another study 49 drowned children for whom CPR had been initiated by EMS personnel and were admitted to Children’s Hospital of Los Angeles, were investigated [6]. Submersion times were not reported. Of the 49 patients, 29 (59%) died in the hospital one day to three months after admission, 13 (27%) were discharged in vegetative states, and 7 (14%) made good recoveries [6]. Eleven of those 13 vegetative survivors were followed up. Four of the 11 had died and the remaining seven had no significant improvement after the hospital discharge. Extensive neuropsychological testing indicated that the seven children with apparently intact recovery showed nearly normal levels of cognitive functioning, except for one child who already had significant development delays prior to the accident. Four of the seven children had neurological examination. Two of them were normal and two had ataxia and motor and coordination deficits. No significant personality disturbances were detected in any of the seven children [6].

In the study by Bratton et al. 44 children were admitted to PICU after drowning [11]. Of these patients 43 had received CPR at the scene. Although it was not mentioned in their study, some of the children most likely received only bystander CPR because of the high survival rate. Submersion times were not reported. Of the 44 patients, 19 died in the hospital (43%), eight (18%) survived with severe neurologic sequelae, and 17 children (39%) had satisfactory outcome meaning mild or no deficits at discharge from the hospital [11]. Children with ataxia and dysarthria were included in patients with satisfactory outcome. The neurologic status of the17 children with satisfactory outcome were evaluated after a follow-up period of minimum of six months by discussing with the respective child’s primary physician or by medical chart review. Fifteen survivors were classified as normal because they had returned to their pre-accident level. One child with mental retardation before the accident had some attenuation of verbal and motor skills and another child had attention deficit disorder [11].

Forty-eight children for whom advanced life support was initiated at the scene and who required admission to the PICU in Southern Finland, were analyzed [8]. The submersion times ranged from 0.5 – 90 min and the median submersion time was 6.3 min. On arrival of the first EMS unit, 23 victims had spontaneous respiration and circulation, one had respiratory arrest and 28 children (58%) were in cardiac arrest. The neurological status of the survivors were retrospectively evaluated using the POPC-scale from medical charts at one year after the accident [15]. Of all 48 children 29 (60%) survived with mild or no disability compared with 10 (21%) with attempted CPR. Seventeen patients died (35%) and two children survived with severe disability (4%) [8].

The longitudinal profile of a 2.5-year old child, after 66 minutes of submersion in icy cold water in Utah and resuscitation on CPB, indicated a pronounced pattern of broad cognitive difficulties [18]. Although, in the original widely referred case report, the child was reported “recovering completely” [17,18]. Subsequent neuropsychological examination revealed impairment of visual-spatial abilities, mild dyslexic characteristics, dramatic memory impairment, full scale IQ of 85, impulsivity, poorconcentration and difficulty in sequential planning and organization. However, the patient's recent MRI and magneto-electrography were within normal limits [18].

A recent, retrospective analyses of single center outcome of nine hypothermic drowning victims treated with CPB in Southern Finland was reported by Suominen et al. [33]. The median submersion time was 38 min (range, 5–75 min). All nine children were able to be weaned from CPB. Unfortunately, only one child became a long-term survivor with mild to moderate neurological deficit based on the neuropsychological tests performed 3-years after the incident. Four of the children died in the PICU and four children within 4 months after discharge from the hospital [33].

Health-related quality of life (HRQoL) scores were reported for Finnish children who were long-term survivors [41]. Each child had received either bystander or emergency medical service personnel initiated CPR after a drowning incident in childhood [41]. The median interval between the accident and follow-up was 10.3 years (range: 1.8 – 21.8 years). According to results of the questionnaire, a fairly good HRQoL was achieved in the vast majority of patients surviving long-term after a severe drowning incident as a child. However, when the submersion time exceeded 10 minutes the mean HRQoL total score was significantly lower than for those patients with an estimated submersion time of less than 10 minutes [41].

The variability of definitions of the patient population and the outcome measurements make it somewhat difficult to assess and draw absolute conclusions from the studies described above and in Table 1. The patient selection in the studies varied from children who had been drowned for a short time without cardiac arrest to those hypothermic children who were brought to the hospital with ongoing CPR and rewarmed by CPB. Therefore, the survival rates of the study populations varied between 11 and 100 percent [33,40].

The POPC scale was used in a retrospective study by Suominen et al. [8]. This scale classifies quality of life into six categories that range from good overall performance to brain death. However, the POPC and similar scales are considered too crude to assess neurologic outcome, although POPC is recommended by the Utstein guidelines for research on drowning [3-15]. The definition of good outcome often includes patients with normal neurological function or mild neurological impairment at hospital discharge, and children with ataxia and dysarthria were included in patients with satisfactory outcome in the study by Bratton et al. [8,11].

Proper neurological and neurophysiological examinations for drowned children are superior to outcome scales based chart reviews. Unfortunately in three studies and two case reviews, neurological and neurophysiological examinations of either apparently intact survivors or of all survivors were performed between 3 to 40 months after the drowning accident [6,10,18,33,40]. Only some of the patients were at school at the time of neurophysiological testing in these studies, which indicates inadequate follow-up duration in most of the patients. In the case report by Hughes et al. neuropsychological examination was performed 12-years after the accident in a child that had been reported “ as recovering completely” in the original case report [17,18]. Neurophysiological tests revealed a pronounced pattern of broad cognitive difficulties [18]. It is known that after the drowning incident a young child may have grossly intact neurological examinations at a short follow-up, but the long-term cognitive sequelae may not manifest until the child enters school or in early adolescence [18].

According to a recent case report on two adult drowned men, both had permanent problems with divided attention, impaired executive functions and poor verbal fluency 3-years after the accident [42]. Problems in skills such as divided attention and executive functions after hypoxic brain injury cannot be exclusively ruled out before early adulthood [18]. It has also been reported that neonates who were resuscitated in the delivery room had an increased risk of a low IQ score at 8 years of age even when they had not developed symptoms of encephalopathy during the neonatal period [43]. These findings strongly show that gross neurological examination at the time of discharge from the hospital in young children does not reveal all the possible sequelaes related to hypoxic brain injury and the need for long-term follow-up of drowned resuscitated children should be continued till adolescence.

According to a study based on a questionnaire on health-related quality of life, a fairly good HRQoL score was achieved in the vast majority of patients who survived long-term after a severe drowning incident as a child [41]. A recent systematic review on patients surviving after cardiac arrest similarly concluded that quality of life in the majority of the patients is good [2]. Future studies should be conducted to see whether HRQoL scores correlate with cognitive function in the child or adolescent after cardiac arrest. Torgersen et al. found no such correlation between reduced level of cognitive function and self-reported quality of life in adults who survived out-of-hospital cardiac arrest [44].

## Conclusion

Accurate prognosis of a drowned child cannot be predicted from the initial presentation or examinations on arrival to the hospital. The duration of submersion, the need of advanced life support at the site of the accident, the duration of CPR, whether spontaneous breathing and circulation are present on arrival at the ER are important factors related to survival with mild neurological deficits or intact function in drowned children. Data on long-term outcome are scarce. The used outcome measurement methods and the duration of follow-up have not been optimal in most of the existing studies. Proper neurological and neurophysiological examinations for drowned children are superior to outcome scales based chart reviews. There is evidence that gross neurological examination at the time of discharge from the hospital in young children does not reveal all the possible sequelae related to hypoxic brain injury and thus long-term follow-up studies of drowned resuscitated children is strongly recommended.

## Abbreviations

EMS: Emergency medical systems; HRQoL: Health related quality of life; POPC: Pediatric Overall Performance Category Scale; PICU: Pediatric intensive care unit.

## Competing interest

The authors have no conflicts of interest to declare.

## Authors’ contributions

Both authors have equally been involved in drafting the manuscript and revising it critically for important intellectual content; and have given final approval of the version to be published.
